# *Audiology Research* Has a New Look!

**DOI:** 10.3390/audiolres10020011

**Published:** 2020-12-02

**Authors:** Giacinto Asprella Libonati

**Affiliations:** UOSD “Vestibologia E Otorinolaringoiatria” Presidio Ospedaliero “Giovanni Paolo II”, 75025 Policoro, Italy; asprella@tin.it

*Audiology Research* (ISSN 2039-4349) is an international, peer-reviewed, open access journal. It was launched in 2011 and published over the past nine years by PAGEPress Publications [1]. Dr. Gabriella Tognola served as the first Editor-in-Chief [2] and I was honored to assume this role in 2014. 

I am pleased to inform you that MDPI has taken over the publication of *Audiology Research* from PAGEPress this year [3].

The mission of *Audiology Research* is to publish contemporary, ethical, clinically relevant scientific research related to basic science and clinical aspects of the auditory and vestibular system and diseases of the ear. This cutting-edge research can be used by clinicians, researchers, scientists and specialists to improve understanding and treatment of patients with audiological and neurotological disorders. The journal publishes original articles, review articles, brief report, case report, comment and reply, conference report, book review, and communication. In addition to publishing regular review and research articles, the journal also publishes “Hot Topic” thematic issues that are guest-edited by scientists in their areas of research.

MDPI editorial offices launched a redesigned website for *Audiology Research*, which is more dynamic and interactive. Together with the redesigned website, an enthusiastic submission requires an energetic and dedicated editorial board to ensure that your article will be reviewed and published rapidly. As Editor-in-Chief I am starting a process of enrichment of the journal by inviting other eminent scientists to join the editorial board to further raise the scientific level and visibility of the journal.

I strongly encourage you to submit your work to be considered for publication in *Audiology Research*. Since the journal is published by a leading publisher, MDPI, a rapid online publication of submitted articles will allow accepted articles to be available to the research community in a truly short time. We will also have special issues by prominent scientists on the most interesting research topics.

We are looking forward to receiving your submissions and welcome your ideas and comments for *Audiology Research*. We look forward to working with our editorial team, our reviewers, and authors, and the MDPI publication team to ensure the growth and success of *Audiology Research*. These administrative and editorial initiatives are meant to improve the management of the journal, to strengthen its editorial perspective (by reinvigorating its role within the discipline as a whole and by including new material in its pages), and to expand its readership.
